# Current Knowledge on Polyethylene Terephthalate Degradation by Genetically Modified Microorganisms

**DOI:** 10.3389/fbioe.2021.771133

**Published:** 2021-11-30

**Authors:** Aneta K. Urbanek, Katarzyna E. Kosiorowska, Aleksandra M. Mirończuk

**Affiliations:** Department of Biotechnology and Food Microbiology, Wrocław University of Environmental and Life Sciences, Wrocław, Poland

**Keywords:** plastic degradation, genetic engineering, microorganisms, PET, protein

## Abstract

The global production of polyethylene terephthalate (PET) is estimated to reach 87.16 million metric tons by 2022. After a single use, a remarkable part of PET is accumulated in the natural environment as plastic waste. Due to high hydrophobicity and high molecular weight, PET is hardly biodegraded by wild-type microorganisms. To solve the global problem of uncontrolled pollution by PET, the degradation of plastic by genetically modified microorganisms has become a promising alternative for the plastic circular economy. In recent years many studies have been conducted to improve the microbial capacity for PET degradation. In this review, we summarize the current knowledge about metabolic engineering of microorganisms and protein engineering for increased biodegradation of PET. The focus is on mutations introduced to the enzymes of the hydrolase class—PETase, MHETase and cutinase—which in the last few years have attracted growing interest for the PET degradation processes. The modifications described in this work summarize the results obtained so far on the hydrolysis of polyethylene terephthalate based on the released degradation products of this polymer.

## Introduction

Worldwide plastic production reached 348 million metric tons in 2017, and this number increases annually by ∼5% (PlasticEurope, 2019; Brahney et al., 2020). Predictions about plastic waste accumulation in ecosystems suggest that in 2050 cumulative plastic waste production will reach over 25 billion tonnes, i.e., 3 times the current level (Geyer et al., 2017). The high resilience and persistence of plastic, previously considered an advantage, nowadays leads to the uncontrolled accumulation of waste in every ecosystem on the planet. Most plastics never completely disappear and only get fragmented into smaller pieces. The formed microplastics (1 μm—5 mm) and nanoplastics (<1 µm) spread all over the globe, reaching pristine regions separated from human activity. For instance, plastic particles have been found in the Arctic Polar Circle (Cózar et al., 2017), Antarctica (Waller et al., 2017), the high mountains (French Pyrenees) (Allen et al., 2019), the Mariana Trench (Gangadoo et al., 2020) and even in the rain in protected areas (Brahney et al., 2020). Easily transported microplastics are extremely dangerous to marine and seacoast animals. It is estimated that more than 800 animal species are affected by plastic waste, and around 90% of all seabirds ingest plastic (Wilcox et al., 2015). Both nanoplastics and microplastics were found in zooplankton and phytoplankton (Rummel et al., 2017), which are consumed by organisms from higher levels of the food chain. Hence microplastics are consumed and accumulated by invertebrates (Thompson et al., 2004). Moreover, it was shown that nanoplastics may reduce the survival of aquatic zooplankton and penetrate the blood-brain barrier in fish and cause behavioural disorders (Mattsson et al., 2017). A recent study showed that crop plants are capable of effective uptake of microplastic and its transport from the roots to the shoots (Li et al., 2020). As it turns out, the ubiquitous plastics also affect the human body. The presence of microplastics was found in the lungs (Pauly et al., 1998) and faecal samples (Schwabl et al., 2019). *In vitro* studies have demonstrated the ability of microplastics to induce an immune response, oxidative stress, cytotoxicity, alteration of membrane integrity and variation in gene expression (Maeza et al., 2021).

Most of the produced plastic material has a fossil origin. Thermoplastic materials such as polyethylene (PE), polyurethane (PUR), polyvinyl chloride (PVC), polypropylene (PP), polystyrene (PS) and polyethylene terephthalate (PET) represent 80% of total global plastic usage (PlasticEurope, 2019). One of the most popular plastic materials used for packing (such as the production of bottles) is PET. PET is a polar, linear polymer of repeating units of aromatic terephthalic acid (TPA) and ethylene glycol (EG). The PET monomer is designated bis(2-hydroxyethyl) terephthalate (BHET). Owing to excellent mechanical and thermal properties, PET is mainly used for beverage bottles, foil, textile fibres and food containers (Danso et al., 2019; Taniguchi et al., 2019; Hiraga et al., 2020). The global production of PET reached 33 million metric tons in 2015 (Geyer et al., 2017) and is still increasing. The problem that has arisen with such enormous production of PET is partially solved by recycling. The main goal of recycling is to obtain new PET or recover the primary components such as TPA and EG so that they can be used as feedstock (Lange 2002). Nowadays, the recycling of PET is mainly based on chemical and mechanical methods. For instance, the mechanical recycling method for PET, melt extrusion, results in the production of rPET fibres from PET bottle waste (Park and Kim 2014), whereas the most common chemical method, glycolysis, degrades PET to BHET with a yield as high as 95% (Imran et al., 2013; Liu et al., 2020). Although these methods are commonly used, they still have some limitations such as spontaneous degradation during the lifetime of new PET obtained after re-extrusion (Park and Kim 2014) or requirement of high temperature (150–300°C) and catalysts in the glycolysis reaction. Especially using catalysts (metal-based, organic or ionic liquids) leads to the high cost of reagents and methodologies, a negative environmental impact and sometimes to the limitation to small-scale trials of reactions (Liu et al., 2020, Sang et al., 2020). In recent years, biological methods have been developed alongside the chemical and mechanical methods of PET recycling. Biological methods are promising and eco-friendly solutions for the decomposition of PET waste. Although PET is labelled as non-biodegradable, much research succeeded in the use of microorganisms or enzymes to break it down. A flagship example is the discovery of the bacterium *Ideonella sakaiensis* 201-F6 and the enzymes PETase and MHETase (Yoshida et al., 2016; Furukawa et al., 2019), which are the focus of many scientists due to very promising aspects of future management of PET. Other enzymes such as cutinases Thc_Cut1 and Thc_Cut2 from *Thermobifida cellulosilytica* DSM44535 (Acero et al., 2011), cutinase FsC from *Fusarium solani pisi* (Egmont and de Vlieg, 2000), cutinase HiC from *Humicola insolens* and lipase CALB from *Candida antarctica* (Carniel et al., 2017) or cutinase TfH from *Thermobifida fusca* DSM43793 (Müller et al., 2005) are also the subject of numerous studies. To date, scientists have verified 27 enzymes that degrade synthetic polymers (Danso et al., 2019), among which enzymes involved in the degradation of PET are typical serine hydrolases, e.g., cutinases (EC 3.1.1.74), lipases (EC 3.1.1.3), and carboxylesterases (EC 3.1.1.1) (Roth et al., 2014). Despite the knowledge of many enzymes, there are many unsolved issues regarding their practical use to degrade PET such as low thermal stability or transfer on an industrial scale (Sang et al., 2020). Much more investigation is needed for mutational developments of the enzyme’s active site, which may help to overcome the limitations.

The degradability of the polymer depends on different factors such as shape, size, presence of various substituents, e.g., chloride atoms or benzene rings, and it decreases with the increase in the molecular weight (Kumari et al., 2019; Rose et al., 2020). The bottlenecks in plastic biodegradation are their high hydrophobicity, crystallinity, strong chemical bonds and high molecular weight (Urbanek et al., 2018). In the past years, a number of studies have been conducted in order to show that many microorganisms and enzymes are capable of degrading plastic. Researchers were mostly focused on the biodegradation performed by wild-type strains, isolated directly from different environments, especially from plastic contaminated areas.

**FIGURE 1:**
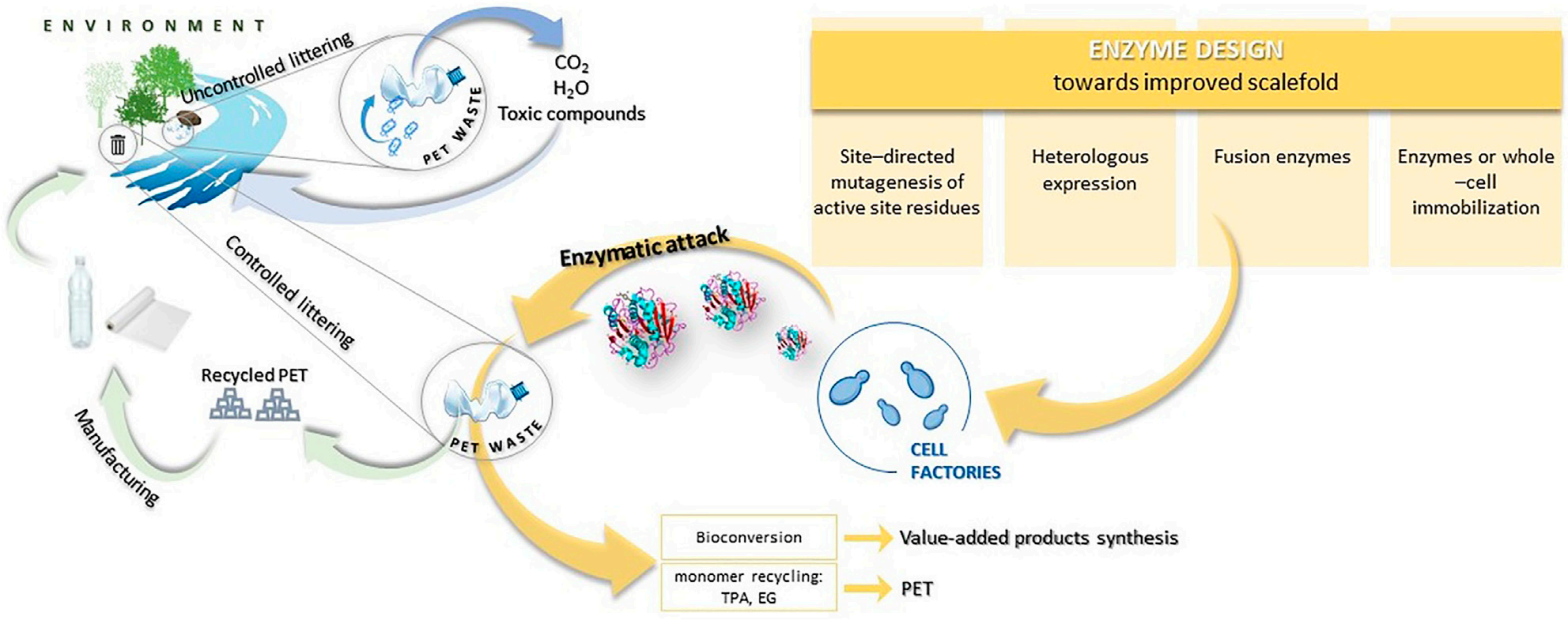
Scheme of plastic circulation in the natural environment and the possibilities of the plastic circular economy.

Although this approach is justified due to the ubiquity of microorganisms and their diverse biodegradability, current studies should be more focused on improving these properties. Published reports show that naturally isolated microorganisms possess a limited capability for plastic degradation. Thus, more efficient production of enzymes and the improvement of enzymes’ activity that would target specific materials with greater selectivity is a key to the improvement of the biodegradation rate of plastic. Employing metabolic engineering provides powerful opportunities in this field (Figure 1).

Here, we present the possibilities of genetic manipulation in order to obtain mutant enzymes with improved catalytic activity and thermostability in the hydrolysis of polyethylene terephthalate (PET) and other polymers.

### Enzymes Involved in PET Degradation

Mostly, the biodegradation of PET is possible by the enzymatic activity of cutinases (EC 3.1.1.74) or PETase (EC 3.1.1.101) with the cooperation of MHETase (EC 2.1.1.102). A number of cutinases with PET biodegradable activity were found, e.g., cutinase from *Humicola insolens* (HiC), *Thermobifida fusca* (TfCut2), leaf-branch compost (LCC) (Tournier et al., 2020), and *Ideonella sakaiensis* (PETase and MHETase) (Yoshida et al., 2016; Furukawa et al., 2019). Cutinases are able to hydrolyse both ester bonds found in aliphatic and aromatic polyesters, hence their wide application in degradation studies of a broad range of plastic polymers (Sulaiman et al., 2012; Liu et al., 2019a). In contrast, PETase can hydrolyse ester bonds present only in aromatic polyesters (Austin et al., 2018). Enzymes involved in PET degradation belong to the esterase subclass and possess a catalytic triad characteristic for α/β-hydrolases (Ser-His-Asp). Ester bond hydrolysis is provided due to the nucleophilic attack by the serine oxygen to the carbonyl carbon present in the ester bond. Negatively charged aspartate stabilizes positively charged histidine residue; thus the established charge transfer network enables serine to carry out a nucleophilic attack (Han et al., 2017). So far, homology has been found in the sequences of cutinases and PETase. Yoshida et al. (2016) observed 51% similarity in amino acid sequence with the hydrolase present in *Thermobifida fusca* (TfH). Furthermore, similar to cutinase from *Fusarium solani*, PETase from *Ideonella sakaiensis* has two disulfide bridges that stabilize the structure of the enzyme molecule. Moreover, as was demonstrated before, an additional disulfide bond in PETase influences the thermal stability of the enzyme (Matak and Moghaddam, 2009; Joo et al., 2018). Phylogenetic analyses performed comparing these two enzymes have also revealed the presence of a highly conserved region recognized as a nucleophilic elbow that contains serine in the central part of the consensus sequence (Joo et al., 2018). Despite the similarities, an important difference is the width of the active site cleft, in comparison with cutinase from TfH; this slot is three times larger at its widest point in PETase (Austin et al., 2018). Furthermore, the residues surrounding the nucleophilic serine in the catalytic triad were found to be considerably different, which affects the substrate selectivity represented by these enzymes (Liu et al., 2018b).

**FIGURE 2:**
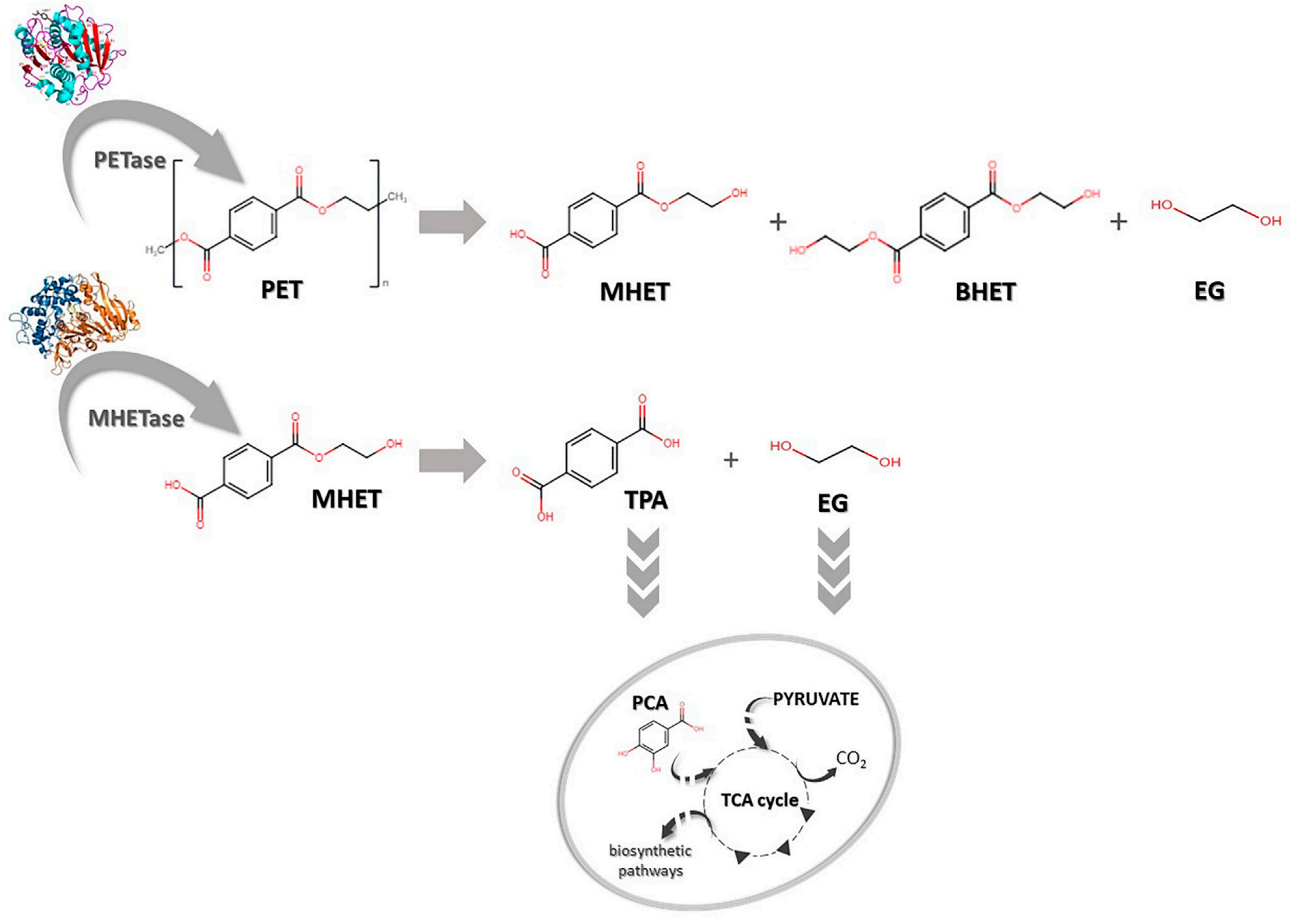
Biodegradation of polyethylene terephthalate (PET). PET can be degraded into mono-(2-hydroxyethyl) terephthalate (MHET), bis-(2-hydroxyethyl) terephthalate (BHET) and ethylene glycol (EG) by PETase. MHET can later be hydrolysed to terephthalic acid (TPA) and EG by MHETase. TPA and EG can be further transported into the cell and converted to protocatechuic acid (PCA) and subsequently integrated into cellular metabolisms via the tricarboxylic acid cycle (TCA).

Recently, many studies have been conducted to improve and better understand the mechanisms of action of these enzymes, especially PETase or MHETase (Oda 2021; Pinto et al., 2021). PETase is recognized as being responsible for hydrolytic conversion of PET into oligomers of mono-2-hydroxyethyl terephthalate (MHET), whereas MHETase hydrolyses MHET into terephthalic acid (TPA) and ethylene glycol (EG) (Figure 2). Thus, recombination and overexpression of those enzymes may be crucial for more efficient degradation of PET as well as monomer recycling (TPA and EG) and for bioconversion to high-value compounds (Furukawa et al., 2019; Taniguchi et al., 2019).

In mechanisms of enzymatic degradation of polyesters, such as PET, apart from plastic properties, the protein structure plays a key role. Especially, the regions on the surface outside the active site of the enzymes and binding modules are essential, both in interaction with the polymer and during the hydrolysis (Acero et al., 2013). For instance, Liu et al. (2018a) assumed in their study that the wide substrate-binding pocket of PETase is critical for PET hydrolysis (Liu et al., 2018b). In contrast, Austin et al. (2018) narrowed the binding cleft and observed improvement in PET degradation (Austin et al., 2018). It should also be emphasized that mutations are frequently used to create greater space in the active sites to fit the large, inaccessible polymer particles and to construct a more hydrophobic substrate-binding site (Araújo et al., 2007). Silva et al. (2011) noted that levels of adsorption to the PET surface are affected by the hydrophobic character of the enzyme active site (Silva et al., 2011). Thus, almost all modifications are related to the active sites of enzymes or their external part (Table 1). Unfortunately, during the creation of enhanced mutants, some obstacles arise. For instance, one of the difficulties emerging during enzymatic degradation of PET by PETase is the location of the enzyme inside the cells. It is perceived as a limiting factor in direct contact of PETase with the solid PET. The consequence is difficulty in establishing a high-throughput screening method in the evaluation of the hydrolysis rate by the modified strains. Fortunately, the solution is an application of developed cell-free protein-expression systems. The system is known as a useful tool in functional and structural proteomics for proteins that could not be expressed *in vivo* in bacterial cells. Furthermore, the system offers several advantages in comparison to traditional cell-based expression methods. First of all, it allows for easy modification of reaction conditions, shortening expression time or reducing the volume of reaction. The productivity exceeds hundreds of micrograms of protein per millilitre of reaction volumes. Interestingly, in the light of the PET problem mentioned above, application of a cell-free protein-expression system allows for the direct contact of expressed protein with solid PET, providing high-throughput screening of PET hydrolytic enzymes (Murthy et al., 2004; Katzen et al., 2005; Ma et al., 2018).

**TABLE 1:** Genetic modifications of enzyme structure for enhancement of biodegradable abilities towards plastic.

Wild-type enzyme/strain	Wild-type microorganisms	Substrate specificity	Vector and host	Improvement in activity	Mutatation’s information	References
PETase	*Ideonella sakaiensis*	PET	Plasmid: pET28a; Host: *E. coli* BL21 (DE3)	⁃R61A: 1.6 fold ⁃L88F: 2.0 fold ⁃I179F: 15.0 fold^a^	mutagenesis of six key residues around the substrate-binding groove in order to: create space around the active site; increase the hydrophobicity of the amino acids around the active site; improve the affinity of the amino acids around the active site for PET	Ma et al. (2018)
PETase	*Ideonella sakaiensis*	PET	Plasmid: pET-21b; Host: *E. coli* BL21-CodonPlus (DE3) RIPL	⁃S131A: not detected ⁃D177A: not detected ⁃H208A: not detected ⁃W130A: increased ⁃W130H: increased ⁃M132A: decreased ⁃W156A: decreased ⁃A180I: no change marked ⁃Q90A: decreased ⁃S185H: increased ⁃S209F: decreased ⁃W68L: decreased ⁃Q153L: no change marked ⁃R94A: decreased ⁃N212A: decreased^b^	structure-guided site-directed mutagenesis in: the active sites; substrate binding pockets; the residues involved in stabilizing the rigidity of the active site	Liu et al. (2018a)
PETase	*Ideonella sakaiensis* 201-F6	PET; PEF	Plasmid: pET-21b(+); Host: *E.coli* C41(DE3)	⁃S238F/W159H: 4.13% higher^c^	site-directed mutagenesis to narrow the PETase active site: S238 to provide new *π*-stacking and hydrophobic interactions to adjacent terephthalate moieties: His159 to allow the PET polymer to sit deeper within the active-site channel	Austin et al. (2018)
PETase	*Ideonella sakaiensis* 201-F6	PET	Plasmid: pET32a; Host: *E. coli* XL1-Blue	⁃S131A: decreased ⁃R103G: decreased ⁃C174S: decreased ⁃C210S: decreased ⁃W156A: decreased ⁃S185H: decreased ⁃I179A: decreased ⁃W130A: decreased ⁃W130H: decreased ⁃M132A: decreased ⁃Y58A: 80.73% MHET production; TPA production decreased ⁃T59A: full activity in producing MHET; TPA production decreased^d^	site-directed mutagenesis to determine apo- and complex crystal structures of PETase and to identify key residues requires for catalysis by, for instance, disruption intra-molecular disulfide bridges DS1 or substitution His residue in the corresponding position	Han et al. (2017)
PETase	*Ideonella sakaiensis*	PET	Plasmid: pET15b; pET15a; Host: *E. coli* Rosetta gami-B	⁃S160A: almost complete loss ⁃D206A: almost complete loss ⁃H237A: almost complete loss ⁃Y87A: 5% hydrolytic activity ⁃M161A: 52% hydrolytic activity ⁃W185A: 5% hydrolytic activity ⁃I208A: 46% hydrolytic activity ⁃W159A: 8% hydrolytic activity ⁃S238A: similiar hydrolytic activity ⁃N241A: 18% hydrolytic activity ⁃R280A: similiar hydrolytic activity ⁃W159H: dramatically decreased ⁃S238F: dramatically decreased ⁃C203A/C239A: dramatically decreased^e^	structural and site-directed mutagenesis in order to confirm the residues involved in enzymatic catalysis and substrate binding: three catalytic residues S160, D206 and H237 replacement with A; four subsite I residues Y87, W185, M161 and I208 replacement with A; three subsite II residues W159, S238, and N241 replacement with A, W159 and S238 residues replacement with H and F; deletion of additional disulfide bond	Joo et al. (2018)
cutinase Thc_Cut2	*Thermobifida cellulosilytica* DSM44535	PET	Plasmid: pET26b(+); Host: *E. coli* BL21-Gold(DE3)	⁃R19S: 3.4 fold ⁃R29N: 17.6 fold ⁃A30V: 17.0 fold ⁃Q65E: decreased ⁃L183A: 1.4 fold ⁃R187K: 4.9 fold ⁃double mutant R29N A30V: 8.4 fold ⁃triple mutant R19S R29N A30V: 7.2 fold^a^	site-directed mutagenesis of amino acids located outside the active site on the Thc_Cut2 surface—exchange of selected side chains with the corresponding side chains of more active Thc_Cut1	Herrero Acero et al., 2013
cutinase	Fusarium solani pisi	PET	Plasmid: pET25b(+); Host: *E. coli* BL21 (DE3)	⁃L81A: 4.0 fold ⁃L182A: 5.2 fold ⁃N84A: 1.7 fold ⁃V184A: 2.0 fold ⁃L189A: decreased^f^	site-directed mutagenesis to create more space in the active site of the cutinase	Araújo et al. (2007)
cutinase Tfu_0883	*Thermobifida fusca*	PET	Plasmid: pET20b; Host: *E. coli* BL21 (DE3)	⁃I218A: 1.2 fold ⁃Q132A/T101A: 1.6 fold^f^	site-directed mutagenesis to create space and to increase hydrophobicity of the catalytic side	Silva et al. (2011)
cutinase TfCut2	*Thermobifida fusca* KW3	PET	Plasmid: not mentioned; Host: *E. coli* BL21 (DE3)	⁃G62A: 4.0 fold	exchange of selected amino acid residues of active site in a substrate binding groove of TfCut2 with those present in cutinase LCC	Wei et al. (2016)
cutinase-type polyesterase (Cut190)	*Saccharomonospora viridis* AHK190	PET	Plasmid: pGEM-T; pQE80L; Host: *E. coli* DH5α; *E. coli* Rosetta-gami B (DE3)	⁃S226P: 1.4 fold ⁃S226P/R228S: 2.1 fold ⁃S226P/R228S/T262K: 2.2 fold^g^	cloning a putative cutinase gene (cut190); site-directed mutagenesis to substitute: S226 with P and R228 with the neutral S, T262 with K to enhance the salt-bridge formation	Kawai et al. (2014)
LC-cutinase	leaf-branch compost	PET	Plasmid: pHK; Host: *E. coli* DH5α; *E. coli* BL21 (DE3)	⁃*C. thermocellum* DSM1313:pHK-LCC: 62%^h^	insertion of the signal peptide sequence of cellulose Cel48S and a constitutive promoter of gene Clo1313_2638 (P_2638_) to *Clostridium thermocellum* for the secretory production of LCC	Yan et al. (2020)
LC-cutinase	leaf-branch compost	PET	Plasmids: pET21b(+); pET26b(+); Host: *E. coli* BL21 (DE3*)*	⁃WCCG: 90% in 10.5 h ⁃ICCG: 90% in 9.3 h^i^	site-specific saturation mutagenesis in the first contact shell of groove; replacing the divalent-metal-binding site with a disulfide bridge; mutations to improve thermostability	Tournier et al. (2020)
LC-cutinase	leaf-branch compost	PET	Plasmid: PET28; PJ912; Host: *E. coli* BL21 DE3; *P. pastoris*	⁃LCC-NG ⁃LCC-G: induction of aggregation 10°C higher than LCC-NG; improvement in the catalytic performance for PET hydrolysis; the rate of aggregation was found to be slower	site directed mutagenesis to introduce three putative N-glycosylation sites to improve LCC resistance for aggregation	Shirke et al. (2018)
cutinase TfCut2, LC-cutinase, carboxyl esterase TfCa	*Thermobifida fusca* KW3	PET	Plasmid: pET-20b(+); Host: *E. coli* BL21(DE3)	⁃TfCa/LCC: 47.9% weight loss/24 h ⁃TfCut2/LCC: 20.4% weight loss/24 h^j^	site-directed mutagenesis for immobilization of TfCa on SulfoLink resin by addition an oligopeptide of G-S-C at the C-terminus of TfCa	Barth et al., 2016; Oeser et al., 2010
cutinase (Cut) and lipase (Lip)	*Thermomyces lanuginosus* (Lip); *Thielavia terrestris* NRRL 8126 (Cut)	PVAC; PCL	Plasmid: pPICZαA; Host: *E.coli* DH5α; *P. pastoris* KM71H	⁃Lip-Cut: 13.3, 11.8 and 5.7 times higher compared to Lip, Cut and Lip/Cut mixture, respectively	construction of chimeric lipase-cutinase (Lip-Cut) system overexpressed in *P. pastoris* to enhance the synergistic action of both enzymes	Liu et al., 2018b; Liu et al., 2019b
cutinase 1 (Thc_Cut1)	*Thermofida cellulosilytica*	PET, PBS, PHBV	Plasmid: pMK-T; pPICZαB; Host: *E. coli* XL-10 cells; *P. pastoris* KM71H	⁃Thc_Cut1_koAsn: no significant differences (PET)^d^ ⁃Thc_Cut1_koST: no significant differences (PET)^d^; 92% of weight loss (PBS)^k^	knock out of the three glycosylation sites at N29, N49, N161 (Thc_Cut1_koAsn) and S31, T51, S163 (Thc_Cut1_koST) by changing the nucleotide sequence to investigate the influence of glycosylation on the activity and stability	Gamerith et al. (2017)
polyhydroxybutyrate depolymerase (PA_PBM) and polyamidase (PA)	*Alcaligenes faecalis* (PA_PBM); *Nocardia farcinica* IMA 10152A (PA)	PUR	Plasmid: pET26b(+); Host: *E. coli* XL10-Gold; *E. coli* BL21	⁃fusion polyamidase PA_PBM: 4 fold	C-terminal fusion of a hydrophobic binding module of PA_PBM to PA to target the catalytic domain to the polyester interface more effectively	Gamerith et al. (2016)
Alkane hydroxylase	*Pseudomonas* sp. E4	LMWPE	Plasmid: pUC19; Host: *E. coli* BL21	⁃recombinant cell viable even after the biodegradation tests at 37 °C for 80 days	expression of alkane hydroxylase gene (*alkB*) in *E.coli* BL21 to mineralize LMWPE	Yoon et al. (2012)

a expressed by kinetic parameters (kcat/KM).

b expressed by PET, degradation efficiency towards PET, bottle.

c expressed by the loss in the absolute crystallinity.

d expressed by the production levels of MHET, and TPA.

e expressed hydrolytic activity using BHET, as a substrate.

f expressed by released TPA, during hydrolytic activity towards PET.

g expressed as enzyme activity measured under standard conditions.

h expressed by the weight loss of PCL, films.

i expressed as enzymatic depolymerization of post-consumer PET, waste.

j expressed by PET, degradation efficiency towards PET, films.

k expressed by the weight loss of PBS, films.

### Engineering of PETase

In 2016 Yoshida et al. published a report about the newly isolated bacterium *Ideonella sakaiensis* 201-F6 that was able to use PET as its major carbon and energy source (Yoshida et al., 2016). Because knowledge of the protein structure is crucial for its further modifications, shortly afterwards many reports about *I. sakaiensis* PETase (IsPETase, EC 3.1.1.101) structure were published. It was shown that this enzyme is a hydrolase and possesses a strictly conserved active site with a Ser-His-Asp catalytic triad and contains an optimal substrate binding site to hold four mono(2-hydroxyethyl) terephthalate (MHET) moieties of PET. PETase enzyme exhibits an optimum pH range of 7–9 and the stability between pH 6 and 10 (Liu et al., 2019b). For purified PETase enzyme applied on PET film, pH 9.0 was identified as optimal, whereas the optimum temperature was estimated as 30°C (Han et al., 2017). PETase exhibits lower activity on *p*-nitrophenol-linked aliphatic esters in comparison to other cutinases, but towards PET the enzyme exhibits 5.5- to 120-fold higher activity compared to the other enzymes (Han et al., 2017; Joo et al., 2018). Attempts to improve the native PETase enzyme from *Ideonella sakaiensis*, which requires a mild environment for growth, are motivated by the relatively low stability of this enzyme. Introducing modifications to the amino acid chain may result in enhanced thermal stability by this protein and could help it maintain activity for a longer time (Joo et al., 2018). Mostly the enzyme’s improvement is focused on site-directed mutagenesis. In the study of Joo et al. (2018) among 14 mutants, created by structural and site-directed mutagenesis, only the variant IsPETase^R280A^, where the arginine (R) in position 280 was replaced with alanine (A), showed increased activity of PETase. The activity towards PET film as a substrate increased by 22.4% in 18 h and 32.4% in 36 h in TPA and MHET release in comparison to IsPETase^W/T^. This mutant also expressed hydrolytic activity using BHET as a substrate at a similar level compared to the wild-type PETase (Joo et al., 2018).

The subsequent study focused on analysing the structure of the PETase enzyme molecule, comparing it to other ɑ/β-hydrolases enzymes, and performing the most promising modifications that could affect the thermal properties of this protein (Son et al., 2019). The possibility for enhancement of the PETase enzyme was the introduction of two mutations that, as previously, would allow the establishment of additional hydrogen bonds to stabilize the molecule. For this purpose, an IsPETase variant possesses changes in serine (S) located at position 121 (to aspartic acid (D) or glutamic acid (E)) and aspartate (D) (to histidine (H)) in position 186 resulting in the S121D/D186H and S121E/D186H mutants have been established.

In other studies, Son et al. (2019) have applied a previous achievement of generating the PETase R280A mutant in the work of Joo et al. (2018) and introduced it to the S121E/D186H mutant described above. The study showed that the obtained triple mutant (S121E/D186H/R280A) degrades PET 13.9-fold better than the native protein and 2.3-fold than the previously established R280A protein variant.

Next, Dai et al. (2021) proceeded with further prospectively profitable changes to the structure of this protein variant. The changes in the enzyme were based on the addition of hydrophobic substrate-binding domains such as CBM (cellulose-binding domain), PBM (poly(3-hydroxybutyrate)) binding domain and HFB4 (hydrophobin) to the C-terminus end of PETase. Authors supposed that the presence of CBM, PBM or HFB4 domain in the protein structure could improve the enzyme binding to the hydrophobic surface of PET molecules, which would be associated with an enhanced level of plastic degradation by these mutants. The effect of the implemented modifications was tested based on the amount of PET degradation products (TPA and MHET) released during the incubation with the novel mutants compared to IsPETase D121E/D186H/R280A (IsPETaseEHA). The experiments showed that among the three obtained mutants (IsPETaseEHA_CBM, IsPETaseEHA_PBM, IsPETaseEHA_HFB4), only the protein containing an additional CBM domain improves PET degradation. Increase in PET breakdown products concentration was 2.28-fold increased in comparison to the original mutant and was 251.5 µM of total hydrolysis products. The two remaining variants significantly reduced PET degradation capacity (Dai et al., 2021).

In the study Han et al. (2017) created 12 mutants in order to identify key residues required for catalysis, most of them showed decreased activity in production levels of MHET and TPA compared to the wild-type PETase. Only variant Y58A (possessing change in tyrosine (Y) at position 58 to alanine (A)) exhibited 80.73% MHET production compared to MHET released by wild-type PETase and T59A, which showed full activity in producing MHET. However, in both cases, TPA production decreased (Han et al., 2017). Ma et al. (2018) aimed to create novel high-efficiency PETase mutants through mutagenesis of six key residues around the substrate-binding groove of PETase. By application of a rapid cell-free screening system, they obtained three mutants. In comparison with wild-type PETase, the R61A (exchange in arginine (R) to alanine (A)), L88F (leucine (L) in position 88 changed to phenylalanine (F)), and I179F (isoleucine (I) exchanged to phenylalanine (F)) mutants exhibited 1.4, 2.1 and 2.5 fold increases in the enzymaticaffinity to PET, respectively. The strongest catalytic activity expressed by TPA concentration and by weight loss of PET film incubated with purified enzyme was shown by the I179F mutant (6.38 mmol L^−1^ of released TPA after 48 h of incubation and 22.5 mg per μmol·L^−1^ PETase per day). L88F and R61A mutants reached 17.5 and 13.5 per μmol·L^−1^ PETase per day, respectively, whereas the degradation rate of wild-type PETase was only 8.2 mg per μmol·L^−1^ PETase per day. Furthermore, scanning electron microscopy (SEM) was used to observe the changes in the morphology of the PET film surface after treatment with the I179F mutant in comparison to the negative control. The surface of the PET film was roughened and eroded, and a large number of holes were observed (Ma et al., 2018). In the study of Liu et al. (2018b) structure-guided site-directed mutagenesis was used to improve PETase catalytic efficiency. Several mutants were created with mutations in the active sites, substrate binding pockets or in the residues involved in stabilizing the rigidity of the active site. The hydrolytic activity of PETase was analysed with respect to BHET. Only two mutants described as W130H, where tryptophan (W) has been replaced by histidine (H) and S209F possessing serine (S) exchange to phenylalanine (F), showed increased activity. Interestingly, the authors performed a PETase activity assay on PET drinking bottles. Similarly, only two mutants, described as W130A and W130H, had higher hydrolytic activity towards PET bottles in comparison to unmodified PETase (Liu et al., 2018a). However, PETase retains the ancestral α/β-hydrolase fold with a core consisting of eight *ß*-strands and six *α-*helices and exhibits a more open active-site cleft than cutinases. Austin et al. (2018) narrowed the binding cleft *via* site-directed mutagenesis of two active-site residues and surprisingly observed improved PET degradation. They created the double mutant S238F/W159H (with serine (S) in position 238 replaced with phenylalanine (F) and tryptophan (W) in position 159 exchanged to histidine (H)) that altered important substrate-binding interactions. The S238 mutation provided new *p*-stacking and hydrophobic interactions to adjacent terephthalate moieties, while the conversion to His159 from the bulkier Trp allowed the PET polymer to sit deeper within the active-site channel. Moreover, in the study, it was demonstrated that the mutant could degrade polyethylene-2,5-furandicarboxylate (PEF), which is a PET replacement. The results suggested that PETase is not fully optimized for crystalline PET degradation (Austin et al., 2018).

Another modification of PETase was performed using the Premuse tool(Meng et al., 2021), by which the selected putative mutations in the protein structure could correspond to natural future evolution in the amino acid chain of the protein. A thorough in-silico analysis highlighted the potential positive effect of the W159H/F229Y mutation to boost the catalytic capacity of PETase. The newly obtained PETase double mutant having modified tryptophan (W) at position 159 to histidine (H) and phenylalanine (F) at position 229 to tyrosine (Y) showed higher thermal stability compared to the wild-type enzyme and the single variants of the mutant proteins (W159H and F229Y). The authors indicated that IsPETase W159H/F229Y after 24 h reaction at 40 °C resulted in a 40-fold increased amount of degradation products in comparison with the native enzyme, however, the authors did not report the specific values of the obtained concentrations of the released compounds (Meng et al., 2021).

### Engineering of MHETase

The MHETase discovery occurred at a similar time as PETases, but it is not as well studied an enzyme as PETase despite the fact that they are cooperatively responsible for the degradation of PET by Ideonella sakaiensis 201-F6 (Tanasupawat et al., 2016). Structurally, MHETase is an α/β hydrolase that exhibits high substrate specificity and its catalytic triad is formed by S225-H528-D492. Additionally, the domain arrangement is similar to those observed in feruloyl esterases but in contrast to them, MHETase exists as a monomer instead of a dimeric structure (Sagong et al., 2020). MHETase possesses optimum temperature at 45°C and a wide range of pH activity between 6.5–9.0 (Palm et al., 2019). Similar to other hydrolases, MHETase performs a nucleophilic attack on the carbonyl carbon via serine (Pinto et al., 2021).

The metabolic engineering of MHETase is not yet as strongly advanced as that of PETase described in detail in the previous section. Nevertheless, we can highlight several examples of previous studies in which modifications in the amino acid sequence of this protein have been undertaken. One of the earliest MHETase mutagenesis was carried out during the work on the determination of its exact structure and involved an amino acid change within the active site of the enzyme. Palm et al. (2019) have generated a number of mutants to identify key amino acid residues in terms of enzyme activity. Their study revealed that one of the key amino acids responsible for substrate binding is Phe495, whose replacement with alanine (A) resulted in the formation of the F495A protein variant. Studies of catalytic properties of this mutant have shown that the turnover rate of MHET compared to the wild-type enzyme was more than 2 times lower and was about 5 s-1 (Palm et al., 2019).

The subsequent study involving engineering MHETase to enable its degradation of BHET was conducted by Sagong et al. (2020). Investigations performed by these researchers indicated that MHETase can bind to BHET as substrate, however, the hydrolysis activity is very low. Studies with targeted mutagenesis indicated an important role of hydrophobic residues Leu254, Trp397, Phe415 and Phe495 in substrate binding and enzymatic catalysis. Sagong et al. (2020) have performed several mutations that significantly affected BHET binding by the created mutants. All of them were based on mutagenesis at phenylalanine position 424 (F424), and for three mutants (F424N, F424V, and F424I), a significant, more than 3-fold increase in activity against BHET relative to native MHETase was observed. Additional arginine point mutation at position 411 to lysine (R411K) was also found to result in a 1.7-fold increase in activity against BHET substrate compared to the wild-type enzyme. Based on the results, further mutagenesis was performed incorporating the revealed properties of the single mutants, which resulted in the formation of double protein variants (R411K/F424N, R422K/F424V, and R411K/F424I). The relative activity to BHET for the resulting mutants was 8.7, 10.5 and 11.1%, respectively than the native MHETase possessing 1% relative activity towards this substrate. Further studies on the MHETase mutants were conducted based on a previous report by Palm et al. (2019) in which an important role for the S416A mutation was identified. The resulting R411K/S416A/F424I triple mutant was shown to be 15.3-fold more active against BHET than wild-type MHETase. Activity assays against amorphous PET film were performed on the triple mutant in two variants: without prior hydrolysis of IsPETaseEHA and after incubation with the modified PETase enzyme (see the paragraph on PETase protein engineering above for a detailed description of this mutant). As expected, neither the wild-type MHETase nor the enhanced triple mutant showed activity against PET films without prior IsPETaseEHA pre-treatment. Interestingly, the researchers found that with the use of PET film pre-treated for 10 days with IsPETaseEHA, both the wild-type MHETase enzyme and the triple mutant R411K/S416A/F424I showed activity against PET film. Specified values obtained in this study after 72 h was about 8 µM of released degradation products by mutant variant, while for the control (wild-type MHETase) it was 4 µM (Sagong et al., 2020).

### Modification of Cutinases

Cutinases (EC 3.1.1.74) are similar to PETase. They belong to the α/β hydrolases group and possess the classical catalytic triad Ser-His-Asp. In nature, cutinases are produced by plant pathogens to hydrolyse the polyesters of the cutin and the suberin layers. In addition, cutinases are able to catalyse reactions with various polyesters and other substrates such as long-chain triacylglycerols or waxes (Nyyssola, 2015). Cutinases possess a wide spectrum of pH optima, where most prefer neutral or alkaline pH. For the thermophilic bacteria Thermobifida fusca, researchers indicate a range of pH at 6.8–9 with optimum pH at 8.0 at an optimum temperature of 50–55 °C (Acero et al., 2011; Hegde and Veeranki, 2013). In the case of fungal cutinase using the example of cutinase from Fusarium solani, the optimum enzyme condition was determined in the range of pH 7.5–10 (Chen et al., 2008; Baker et al., 2012) and the optimum temperature range for this cutinase has been indicated at 25°C (Baker et al., 2012), 30°C (Chen et al., 2008) and 40°C (Pio and Macedo, 2009).

Since cutinases are universal and efficient esterases, their modification toward PET degradation has been done (Acero et al., 2013). The effect of site-directed mutagenesis, which exchanges selected surface-located amino acids between two polyester hydrolases from *thermobifida cellulosilytica* DSM44535, has been studied. As a result, six single mutants, one double mutant and one triple mutant were obtained. The degradation level of amorphous PET films was tested by enzymatic hydrolysis with the use of derived cutinases and quantification of the released degradation products (TA-terephthalic acid and MHET-mono-(2-hydroxyethyl) terephthalate). Incubation of PET with unmodified cutinase Thc_Cut2 as a control was provided. PET hydrolysis was performed for 2 days at 50°C at pH 7.0 with the 200 µgmL-1 of enzyme on pre-washed PET films with Triton-X 100 (Acero et al., 2013). The pre-treatment of non-ionic surfactant used in this study may lead to a decrease in the hydrophobicity of the polymer surface and consequently facilitate the binding of the enzyme with the substrate (Caparanga et al., 2009; Mohanan et al., 2020). Kinetic parameters for the mutants compared to the Thc_Cut2 (9 s-1mM-1) were performed, as a result, mutants carrying Arg29Asn (15 s-1mM-1) and/or Ala30Val (153 s-1mM-1) exchanges showed considerably higher specific activity and higher kcat/KM values on soluble substrates. However, it should be noted that a triple mutant enzyme with Arg19Ser introduction negatively influenced all the parameters (Acero et al., 2013). Experiments performed on PET film, based on the measurement of TA and MHET released during hydrolysis showed that there is no significant increase in MHET concentration. However, an increased amount of TA released during PET degradation compared to Thc_Cut2 occurred for Ala30Val, Arg29Asn_Ala30Val and Arg19Ser_Arg29Asn_Ala30Val mutations. The highest TA concentrations measured in this experiment were 400 and 370 mM for Arg29Asn_Ala30Val and Arg19Ser_Arg29Asn_Ala30Val, respectively. Interestingly, the introduced Gln65Glu mutation resulted in a 36% decrease in the concentration of the amount of breakdown products for 3PET and completely inhibited PET degradation, despite the fact that kinetic parameters did not remarkably differ compared to the Thc_Cut2.

In other studies, a cutinase from *Fusarium solani pisi* was genetically modified to enhance its enzymatic activity. Site-directed mutagenesis targeted the region near the active site and as a result, two mutants with enhanced activity towards polyester fibres were obtained, named L81A and L182A. They showed an activity increase of four- and five-fold, respectively, when compared with the wild type, for PET fibres. The authors explained the increase in activity of these mutations by higher stabilization of TI and better accommodation of the substrate (Araújo et al., 2007).

Another successful improvement of the enzymatic degradation of PET was presented in the study of Silva et al. (2011). The active site of cutinase Tfu_0883 from *Thermobifida fusca* was modified by site-directed mutagenesis to increase the affinity of cutinase to PET and the ability to hydrolase it. The mutation I218A (isoleucine (I) replacement to alanine(A)) was designed to create space and the double mutation Q132A/T101A possessing glutamine (Q) and tyrosine (T) replaced with alanine (A) was designed both to create space and to increase hydrophobicity. The activity of both single and double mutants exhibited considerably higher hydrolysis efficiency towards PET fibres—a double mutant exhibited 1.6-fold increased hydrolysis activity (Silva et al., 2011).

In a similar study conducted by Wei et al. (2016), mutagenesis was used to increase the activity of the cutinase TfCut2 from *Thermobifida fusca*. By exchanging selected amino acid residues of the active site in a substrate-binding groove of TfCut2 with those present in LCC, mutants with increased PET hydrolytic activity were obtained. The most active mutants were G62A, possessing glutamine (G) replaced by alanine (A) and G62A/I213S where additional exchange of isoleucine (I) by serine (S) was done. As a result, a 2.7-fold increase in weight loss of PET films was obtained compared to the wild-type enzyme. Moreover, kinetic analysis based on the released PET hydrolysis products confirmed the superior hydrolytic activity of G62A with a fourfold higher hydrolysis rate constant and a 1.5-fold lower substrate-binding constant than those of the wild-type enzyme (Wei et al., 2016). Next, the mutant TfCut2 G62A obtained by Wei et al. (2016) was a subject of the interesting study of Furukawa et al. (2019). In the study, it was found that low-crystallinity PET (lcPET) hydrolysis may be increased by the addition of a cationic surfactant that attracts enzymes near the lcPET film surface via electrostatic interactions. This approach was applicable to the mutant TfCut2 G62A/F209A and wild-type TfCut2. As a result, the degradation rate of TfCut2 G62A/F209A in the presence of the cationic surfactant (dodecyl trimethyl ammonium) increased 12.7 times over that of wild-type TfCut2 in the absence of the surfactant. A positive effect of surfactant addition was evident for the native enzyme as well as all mutants used except H129E/F209S. The long-duration reaction showed that lcPET film had the fastest biodegradation rate of lcPET film so far (97 ± 1.8% within 30 h) (Furukawa et al., 2019). It was also noted that the addition of a cationic surfactant, as well as the increased reaction temperature, results in enhanced hydrophobic interactions between the enzyme and the plastic surface, and consequently increases the amount of enzyme bound to the lcPET surface (Furukawa et al., 2019). Moreover, higher temperature raises the mobility of the polymer chain, which further facilitates the binding of the enzyme to the substrate (Ronkvist et al., 2009).

Tournier et al. (2020) found that leaf-branch compost cutinase (LCC) demonstrated the highest thermostability and was at least 33 times more efficient than other enzymes tested in their study. In differential scanning fluorimetry experiments it was shown that LCC is thermally stabilized in the presence of calcium ions. To avoid salt supplementation, the authors focused on improving the activity and thermostability of LCC by enzyme engineering. By using the alternative strategy of replacing the divalent metal binding with a disulfide bond the researchers obtained thermal stabilization of LCC without dependence on calcium ions. Moreover, by site-direct saturation mutagenesis, they tested 209 mutants. Most of the modified variants showed less than 1% specific activity in comparison to the wild-type LCC, but the F243I and F243W mutations, in which phenylalanine (F) at position 243 was replaced with isoleucine (I) or tryptophan (W), showed elevated activity. The obtained cutinase variants gained specific activity by 27 and 18%, respectively. Finally, they obtained an enhanced PET hydrolase that was able to depolymerize over 90% PET into monomers in over 10 h (10.5 and 9.3 h for mutants WCCG (F243W/D238C/S283C/Y127G) and ICCG (F243I/D238C/S283C/Y127G), respectively with the use of 3 mg of enzyme per 1 g of PET. The productivity of ICCG mutant was determined at 16.7 g of terephthalic acid per litre per hour at 72°C, which is a 98-fold increase compared to TfCut2 investigated before (Wei et al., 2019). Wild type LCC enzyme achieved only 53% of conversion after 20 h, which corresponds with its lower thermostability in comparison to the mutants. Although X-ray crystallography showed no substantial difference between parental LCC and ICCG, molecular-dynamic simulations revealed that mutations introduced in ICCG facilitated the catalytic binding of 2-HE(MHET)3 compared with parental LCC (Tournier et al., 2020).

Kawai et al. (2014) created a Cut190 (S226P/R228S), a double mutant enzyme for PET degradation. They cloned the cutinase gene (cut190) from *Saccharomonospora viridis* AHK190 and expressed it in *Escherichia coli* Rosetta-gami B (DE3). It was observed that the substitution of Ser226 with Pro and Arg228 with Ser yielded the highest activity and thermostability of the new enzyme. Also, they noted that the presence of the Ca^2+^ ion enhanced the enzyme activity and thermostability in comparison to both the wild-type enzyme and mutant Cut190. Circular dichroism suggested that the Ca^2+^ changes the tertiary structure of Cut190 (S226P/R228S) (Kawai et al., 2014). High-level expression of LCC was also achieved due to insertion of the signal peptide sequence of cellulose Cel48S and a constitutive promoter of the gene Clo1313_2638 (P2638) in *Clostridium thermocellum*. Improved degradation of commercial PET films was observed and maximum weight loss (approximately 62%) was achieved after 14 days of incubation at 60°C (Yan et al., 2020).

Genetic engineering may also be a solution to many problems related to the stability of enzymes. For instance, Shirke et al. (2018) underlined that aggregation is emerging as a major factor that reduces LCC kinetic stability. In its native state, LCC is highly prone to aggregation owing to electrostatic interactions. Since LCC precipitates even at room temperature and low concentrations, the purification and storage of enzymes require salt concentrations that vary with protein concentration. Moreover, efficient PET hydrolysis requires a temperature around 70°C, which is very close to the temperature of LCC structure loss. To overcome these problems, Shirke et al. (2018) proposed the expression of native LCC in *Pichia pastoris*, resulting in the production of glycosylated LCC (LCCG). They introduced three putative N-glycosylation sites, which improved resistance to aggregation even at high-temperature conditions, leading to a 10°C increase in the thermal aggregation point and a significant increase in kinetic stability. Furthermore, glycosylation resulted in improved catalytic PET hydrolysis (Shirke et al., 2018). On the other hand, Gamerith et al. (2017) aimed to investigate the influence of glycosylation on the activity and stability of cutinase 1 (Thc_Cut1) from *Thermobifidia cellulosilytica*. They expressed Thc_Cut1 and two glycosylation site knockout mutants, Thc_Cut1_koAsn and Thc_Cut1_koST, in *P. pastoris*. However, the created mutants hydrolysed aromatic (PET) and aliphatic (PHBV and PBS) polyester powders at very different rates based on quantification of released products by HPLC. Thc_Cut1_koST was the most effective among all enzymes. The highest TPA yield was obtained for Thc_Cut1_koST mutant, which caused hydrolysis of 24% of the starting PET powder amount. Due to the fact that the Thc_Cut1_koST mutant exhibited higher protein production yield in engineered *P. pastoris* yeast, this variant was used in further studies on PHBV and PBS degradation. The authors did not observe significant differences in PHBV degradation between Thc_Cut1 and Thc_Cut1_koST mutants, due to the similar amounts of 3-HBA (3-hydroxybutyric acid) at around 0.5 mM after 96 h of incubation. A similar trend was observed by investigating a PBS film weight loss, where a 92% decrease in the mass of polymer film was obtained for the applied mutant and 41% for Thc_Cut1 within 96 h of hydrolysis (Gamerith et al., 2017).

Although the main focus in genetic engineering of microorganisms and enzymes with biodegradation activity is directed towards PET, some studies present results for other plastics. For instance, the alkane hydroxylase gene (alkB) from *Pseudomonas* sp. E4 was expressed in *E. coli* BL21. A recombinant strain secreted recombinant alkane hydroxylase (AH) and was able to mineralize 19.3% of the low molecular weight polyethylene (LMWPE) to CO_2_ after incubation in the compost for 80 days at 37°C, while the recipient cell was not active at all toward LMWPE biodegradation (Yoon et al., 2012).

### Synergistic Activity of Chimeric Enzymes

Chimeric enzymes, also known as fusion proteins, are proteins formed by combining two or more unrelated genes that originally encoded distinct proteins. The resulting proteins exhibit the attributes of all the proteins used in the fusion and constitute a single, combined molecule. Suitably designed hybrid proteins offer many opportunities due to their wide range of properties and can be used in many fields (Yu et al., 2015). Thus, the application of multiple enzyme systems for the biodegradation of plastic seems to be a very promising solution. It is reasonable to assume that enzymes might be used synergistically with other enzymes in polymer degradation due to the complementary properties of both enzymes in both catalysis pattern and substrate specificity (Liu et al., 2019b). A prime example is the connection of two enzymes in the biodegradation of PET. It is known that during PET degradation, accumulating MHET is an important factor that limits the efficiency of hydrolysis. To avoid this problem, the recombinant expression and purification of TfCut2 from *Thermobifida fusca* KW3 and LC-cutinase (LCC) were proposed. In the study of Barth et al. (2016) the dual system was LCC or TfCut2 combined with immobilized TfCa on the SulfoLink resin—which was generated by the addition of oligopeptide of glycine-serine-cysteine at the C-terminus via site-directed mutagenesis. The introduction of the C-terminal oligopeptide did not cause a significant reduction in its hydrolytic activity against *p-*NPB, BHET and MHET. The immobilized enzyme maintained approximately 94% of its initial activity at 60°C, whereas free TfCa resulted in a complete loss of activity at 55 °C. Moreover, the usage of a dual enzyme reaction system with LCC or TfCut2 caused a 47.9% or 20.4% weight loss, respectively, of the PET films after a reaction time of 24 h (Barth et al., 2016). An artificial chimeric enzyme was also constructed by Liu et al. (2018a). In their study lipase (Lip) from *Thermomyces lanuginosus* and cutinase (Cut) from *Thielavia terrestris* NRRL 8126 were used for the construction of bifunctional lipase-cutinase (Lip-Cut) by end-to-end fusion and overexpression in *Pichia pastoris* (Liu et al., 2018b). Lip-Cut exhibited a more efficient degradation ability towards poly(ε-caprolactone) (PCL). The weight loss of PCL films was 13.3, 11.8, and 5.7 times higher (at 6 h) than those obtained by Lip, Cut and the Lip/Cut mixture, respectively. GC-MS analysis revealed that the main products produced during hydrolysis were 6-hydroxyhexanoic acid and 3-caprolactone. Moreover, SEM analysis showed that the surface of the PCL film became rougher and more holes were observed after 4 h of treatment with bifunctional Lip-Cut than in the case of other enzymes after 48 h (Liu et al., 2019a). Gamerith et al. (2016), using C-terminal fusion, fused a hydrophobic binding module of polyhydroxybutyrate depolymerase (PA_PBM) from *Alcaligenes faecalis* to polyamidase (PA) from *Nocardia farcinica* IMA 10152A. The fusion polyamidase (PA_PBM) indeed resulted in a more active enzyme on commercial polyurethane copolymers as indicated by the release of 4,4′-diaminodiphenylmethane (MDA) and different oligomers (Gamerith et al., 2016).

An interesting improvement of PET degradation was the two-enzyme system described by Knott et al., 2020. In that study, the authors constructed an MHETase: PETase chimeric protein covalently linking the C-terminus of MHETase to the N-terminus of PETase of varying glycine-serine linker lengths (8, 12 or 20 aa residues). All chimeric proteins exhibit improved PET and MHET turnover relative to the free enzymes. Hydrolysis of amorphous PET incubated with 0.25 mg of PETase and 0.5 mg of MHETase per gram of PET resulted in the release of 0.25 mM MHET and TPA by PETase and 0.45 mM MHET when co-incubated with the two enzymes. The use of the MP8, MP12 and MP20 chimeras increased the amount of TPA released threefold (1.4, 1.45, and 1.5 mM, respectively). Interestingly, the chimeric constructs linking the C-terminus of PETase to the N-terminus of MHETase were not capable of expressing the protein (Knott et al., 2020).

Another enhancement of PETase based on the implementation of hybrid proteins to increase its activity and thermal stability was performed by Chen et al. (2021). The work conducted by these researchers involved the addition of an amino acid chain containing glutamic acid (E) and lysine (K) to the C-terminus of the PETase enzyme, which resulted in the formation of different PETase-EK fusion protein variants (5, 10 and 30 kDa). Thermal stability assays performed for the obtained mutants compared to the native PETase showed that each of the obtained proteins exhibited better stability (80% activity after 6 h incubation at 40°C) than the native enzyme (65% activity after incubation under the same conditions). The ability to degrade PET was verified by the number of released degradation products during incubation with amorphous PET film and PET bottle film. The experiment demonstrated that each mutant caused a significantly higher level of PET degradation (on both plastic type materials used) compared to the wild-type enzyme. The best results were obtained with PETase-EK30, which after 6 days of incubation at 40°C resulted in the release of 302.4 and 146.2 µM total MHET and TPA after incubation with amorphous PET film and PET bottles, respectively. The incubation with the native enzyme resulted in the release of 32.8 and 13.9 µM of MHET and TPA for the appropriate substrates, correspondingly. It is worth noting that for the native enzyme, from day 1 of incubation to day 6, the amount of products released did not change significantly. In the case of the mutants, a progressive increase in the amount of breakdown products was noted from day 1 to day 4 of incubation, while between days 4 and 6 the measured concentrations were at equivalent amounts (Chen et al., 2021).

### Prospective Applications of Modified Microorganisms and Engineered Proteins in PET Waste Management

Since 1964 production of plastic has increased twentyfold, but almost 50 years after the introduction of the recycling process, only 14% of plastic packing is collected for reuse. PET used in bottles has the highest recycling rate, but globally only 7% of it is recycled bottle-to-bottle. Most of the plastic products after a single use are landfilled, and 32% escape the collection system to the natural environment (Ellen MacArthur Foundation and World Economic Forum, 2014; http://www3.weforum.org/docs/WEF_The_New_Plastics _Economy.pdf). The growing amount of plastic waste has forced the scientific community to look at this global issue. So far the published reports have shown that naturally isolated microorganisms possess a limited capability for plastic degradation, and it might take decades before microbes adapt to use plastic as a carbon source. The published data on genetically modified microorganisms or chemically engineered enzymes suggest that this direction offers a promising method for plastic waste management. Nowadays, through the chemical engineering of enzymes such as modification of the active site or by introducing new bonds, we can avoid cofactor supplementation with the simultaneous multifold improvement of their activities, reduction of the reaction time and increase of their thermal stability. The latter factor might be crucial since hydrolysis of PET needs a higher temperature than the glass transition temperature (Tg), which is 67–81 °C. A highly interesting approach that can be applied to PET degradation technology in the future is the concept of nano-immobilization of enzymes to improve their tolerance to temperature and pH. The first attempts to use immobilized enzymes have been recently achieved successfully (Jia et al., 2021). Another perspective, which should be included in the discussion, is the possibility of reuse of the products of PET degradation (such as EG and TPA) to synthesize a new PET with similar properties as a virgin PET. This will result in reduced demand for fossil substrates for plastic synthesis and simultaneously may be a branch where the use of immobilized enzymes can bring additional benefits. Prospectively, due to restrictions and concerns over the use of GMOs worldwide, safer and easier to safely handle modified proteins may become an alternative to the long-known bioremediation or biological recycling, which seems to be a distant goal when using GMMs. The application of enzymatic recycling with the use of enhanced mutants of PETase cutinase may be one solution in the future to reduce the level of contamination, and to our knowledge, such application is one of the latest developments in this field (Singh et al., 2021).

An interesting attempt was made by Roberts et al. (2020), where a microbial consortium of *Pseudomonas* and *Bacillus* species was applied to synergic PET degradation. Such a study may bring additional perspective about applying an enzymatic cocktail as a possibility to provide ester bond hydrolysis in PET formulated with engineered hydrolases. Despite the fact that microorganisms able to produce PET-degrading enzymes and capable of growing degradation products as the sole carbon source can be found in nature, the efficiency of PET hydrolysis is generally low. The other perspective that can be taken into consideration in further application attempts of genetic engineering and protein modification methods is related to the successful attempt to obtain microalgae capable of producing PETase enzyme (Kim et al., 2020). The use of an improved mutant of this protein in similar studies could greatly enhance the research conducted in this area and bring interesting adaptive solutions for photosynthesizing eukaryotes.

In the future probably we will be able to use plastic wastes as a low-cost substrate for genetically modified microorganisms to produce value-added products such as enzymes, fatty acids, organic acids and others. As emphasized in this review, genetically modified microorganisms are a promising alternative for the plastic circular economy.

## Conclusion

As the production of plastics is still increasing, enzymatic hydrolysis of PET and other plastic is gaining importance and interest from researchers. This way of degradation of plastic is evaluated as an environmentally friendly, novel strategy for the recycling of post-consumer plastic materials. Thus, in order to better adapt the enzymes to synthetic polymers, the use of genetic engineering may be a key to solving the plastic pollution problem. However, the engineering of novel hydrolases exhibiting highly efficient and specific catalytic properties towards PET materials remains a challenge. All findings presented above may provide further options to obtain effective enzymes for biocatalytic plastic recycling processes.
